# The soybean *GmSNAP18* gene underlies two types of resistance to soybean cyst nematode

**DOI:** 10.1038/ncomms14822

**Published:** 2017-03-27

**Authors:** Shiming Liu, Pramod K. Kandoth, Naoufal Lakhssassi, Jingwen Kang, Vincent Colantonio, Robert Heinz, Greg Yeckel, Zhou Zhou, Sadia Bekal, Johannes Dapprich, Bjorn Rotter, Silvia Cianzio, Melissa G. Mitchum, Khalid Meksem

**Affiliations:** 1 Department of Plant, Soil and Agricultural Systems, Southern Illinois University, 1205 Lincoln Drive RM176, Carbondale, Illinois 62901, USA; 2 Division of Plant Sciences and Bond Life Sciences Center, University of Missouri, Columbia, Missouri 65211, USA; 3 Generation Biotech, Lawrenceville, New Jersey 08648, USA; 4 GenXPro-GmbH, Altenhöferallee 3, 60438 Frankfurt am Main, Germany; 5 Department of Agronomy, Iowa State University, Ames, Iowa 50011, USA

## Abstract

Two types of resistant soybean (*Glycine max* (L.) Merr.) sources are widely used against soybean cyst nematode (SCN, *Heterodera glycines* Ichinohe). These include Peking-type soybean, whose resistance requires both the *rhg1-a* and *Rhg4* alleles, and PI 88788-type soybean, whose resistance requires only the *rhg1-b* allele. Multiple copy number of PI 88788-type *GmSNAP18*, *GmAAT*, and *GmWI12* in one genomic segment simultaneously contribute to *rhg1-b* resistance. Using an integrated set of genetic and genomic approaches, we demonstrate that the *rhg1-a* Peking-type *GmSNAP18* is sufficient for resistance to SCN in combination with *Rhg4*. The two SNAPs (soluble NSF attachment proteins) differ by only five amino acids. Our findings suggest that Peking-type *GmSNAP18* is performing a different role in SCN resistance than PI 88788-type *GmSNAP18*. As such, this is an example of a pathogen resistance gene that has evolved to underlie two types of resistance, yet ensure the same function within a single plant species.

## Abstract (web summary)

Peking-type and PI 88788-type soybeans are both used as sources of resistance against soybean cyst nematode. Here the authors show that in contrast to PI 88788-type resistance, where *GmSNAP18* acts in combination with two neighbouring genes, in Peking-type resistance *GmSNAP18* acts with *GmSHMT08* to confer resistance.

Soybean cyst nematode (SCN), *Heterodera glycines* Ichinohe, one of the most devastating pathogens of soybean, causes more than $1 billion in yield losses annually in the United States alone^1^. Although planting resistant cultivars forms the core management strategy for this pathogen, the mechanism of soybean resistance to SCN is still unknown. Moreover, the genetic diversity of resistance is limited and virulent nematode populations have been identified for most known resistant sources. Therefore, understanding the molecular nature of soybean's resistance to SCN is increasingly important for the long-term management of this devastating nematode disease.

The first *Rhg* genes (for resistance to *H. glycines*) were identified in the early 1960s. Numerous reports are available on the identification and mapping of quantitative trait loci (QTL) in soybeans with underlying resistance to SCN from a variety of different germplasm sources. QTL on chromosomes 18 (*rhg1*) and 8 (*Rhg4*) are the two major resistance QTL that have been consistently mapped and reported in a variety of soybean germplasm. In some accessions, such as plant introduction (PI) 88788, *rhg1* is sufficient to provide resistance against the nematode with the gene itself displaying incomplete dominance^2^. In other cases, such as the soybean cultivar (*cv*.) Forrest, resistance to SCN requires both *rhg1* and *Rhg4* (ref. 3). Brucker *et al*. classified the resistant *rhg1* into two types: *rhg1-a* in Peking-type soybeans and *rhg1-b* in PI 88788-type soybeans^4^. Here we define the susceptible *rhg1* type in Essex and Williams 82 as *rhg1-s*; otherwise, we use *rhg1* to refer to the locus in general.

Resistant soybean lines carrying the *Rhg* resistance genes display an incompatible interaction with the nematode. Although infective juvenile nematodes penetrate the plant root, the feeding cells eventually degenerate causing the nematodes to die before they reach the adult stage. *H. glycines* exhibits genetic variability and nematodes carrying the as yet undefined *ror* (reproduction on a resistant host) alleles are able to survive on resistance cultivars thus causing population shifts in the field associated with monoculture or resistant soybean varieties^5^. Recently, progress has been made in the identification of the major soybean genes underlying SCN resistance at the *rhg1-b* and *Rhg4* loci, and in the development of specific DNA markers for SCN resistance selection. Liu *et al*.^6^ identified and functionally validated a serine hydroxymethyltransferase on chromosome 8 (*GmSHMT08*) as the *Rhg4* gene by high-density genetic mapping, targeting-induced local lesions in genomes (TILLING), gene silencing (virus induced gene silencing (VIGS) and RNA interference) and genetic complementation. Serine hydroxymethyltransferases (SHMTs) are important in one-carbon folate metabolism, but how this particular soybean SHMT functions in resistance to the nematode remains unknown. Cook *et al*. reported that the resistance of soybean *rhg1-b* to SCN is simultaneously mediated by the high copy number of three genes (*Glyma18g02580*, *Glyma18g02590* and *Glyma18g02610*) within a 31 kb PI 88788 *rhg1-b* segment^7^. This segment also includes partial gene sequences for *Glyma18g02570* and *Glyma18g02610*. Direct repeats (2–10) are present in SCN-resistant soybean lines, whereas only one copy is present in SCN-susceptible soybean lines^7^,^8^,^9^. Using the *Glyma18g02590* gene sequences, two specific KASP (kompetitive allele-specific PCR) single-nucleotide polymorphism (SNP) markers were developed to select the *rhg1* resistance alleles and the assay can be used to differentiate Peking-type and PI 88788-type resistance^10^.

Using a positional cloning approach, we identified *rhg1-a* candidate genes within a contrasting 370 kb chromosomal interval between the resistant line Forrest and the susceptible line Essex. We then applied ‘region-specific extraction sequencing' (RSE-Seq), a capture technology developed to enrich a targeted chromosomal segment for genome sequencing^11^,^12^,^13^, to identify SCN resistance genes within the identified 300 kb chromosomal segment carrying the *rhg1* locus. RSE-Seq has been applied successfully to study yeast, human and zebrafish^11^,^12^,^13^,^14^,^15^. The SNPs and insertions and deletions (InDels) of four soybean lines (Essex, Forrest, Peking and PI 88788) were analysed using the Williams 82 genomic sequence as a reference^16^. The analysis of the relevant SNPs and InDels identified the *GmSNAP18* as the strongest candidate gene conferring resistance to SCN at the *rhg1* locus. Genetic complementation analyses of the Forrest *GmSNAP18* confirmed its major role in resistance to SCN. The complementation analyses also showed the Forrest *GmSNAP18* is specifically required for Peking-type resistance, as the resistant *GmSNAP18* allele of PI 88788 or the susceptible *rhg1-s* could not restore resistance in the Forrest (Peking-type) *Rhg4* background.

## Results

### Map-based cloning of *rhg1-a* gene conferring SCN resistance

**Figure 1:**
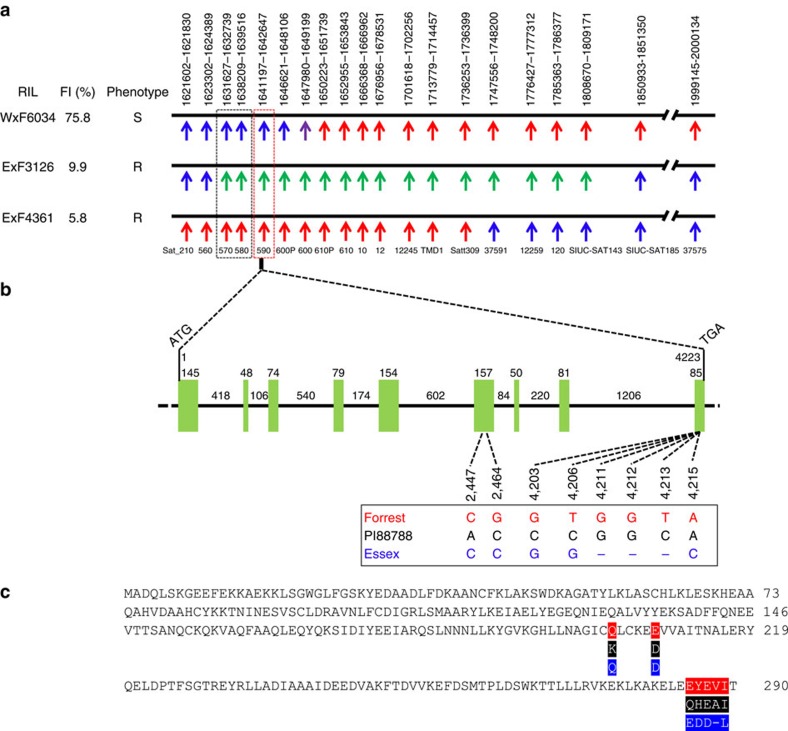
Map-based cloning of the soybean *rhg1-a* gene conferring resistance to SCN. (**a**) The high-density genetic maps of the *rhg1-a* locus of three RILs. The results indicate that the resistant candidates are the region directed by the markers 570, 580 and 590, including 3 genes: *Glyma18g02570*, *Glyma18g02580* and *Glyma18g02590*. The black horizontal lines represent approximately 370 kb of the *rhg1-a* chromosomal interval. The arrows under the black lines designate the position of each DNA marker and its name. Numbers above the black horizontal line denote the genomic position of each marker in Williams 82 genome (http://soybase.org). Blue, red and green arrows represent the markers with Williams 82 or Essex alleles, Forrest alleles and heterozygote alleles (Forrest allele with Essex allele), respectively. Purple arrows represent the marker 600 not showing polymorphisms between Williams 82 and Forrest, but displaying polymorphisms between Forrest/Williams 82 and Essex. (**b**) Gene model for the *GmSNAP18* (*Glyma18g02590*) genomic DNA sequence. The gene is 4,223 bp long and contains nine exons (green boxes) and eight introns (solid black lines). The numbers above the green boxes and the solid black lines indicate the length (bp) of each exon or intron and the numbers under the dotted lines indicate the nucleotide position relative to the first nucleotide of the start codon. Six SNPs and four InDels within the exons were identified. (**c**) Comparison of the predicted *GmSNAP18* protein sequences among Forrest, PI88788 (Type I) and Essex, with the amino acid differences (Q203K, D208E, E285Q, D286Y, D286H, D287E, −288A, −288V and L289I). Red, black and blue represent Forrest, PI 88788 and Essex, respectively, in **b**,**c**.

To perform positional cloning by high-density genetic mapping of the *rhg1-a* gene conferring resistance to SCN, three F_5:6_ recombinant inbred line (RIL) populations segregating for resistance to SCN were developed from crosses of the SCN-resistant *cv.* Forrest (F) with the SCN-susceptible soybean *cvs*. Williams 82 (W) and Essex (E). As Forrest-type resistance to SCN requires both the *rhg1-a* and *Rhg4*, identification of recombinants was conducted using DNA markers flanking both loci to detect informative recombinants at the *rhg1-a* locus (Supplementary Table 1). We identified a total of 222 recombinant lines with chromosomal breakpoints around the *rhg1-a* locus, of which three recombinants (ExF4361, ExF3126 and WxF6034) were crucial in defining the interval carrying the *rhg1-a* or *rhg1-s* gene. All three lines carried the Forrest allele at the *Rhg4* locus; ExF4361 and ExF3126 were resistant, whereas WxF6034 was susceptible to SCN (Fig. 1a). The ExF4361 recombinant suggested that the *rhg1-a* gene(s) was (were) located on the left side of marker 37591. The WxF6034 recombinant indicated that the *rhg1-a* gene(s) was (were) located to the left of marker 600, excluding the DNA marker 600. The ExF3126 recombination event showed that the *rhg1-a* gene(s) was (were) located in the interval between DNA maker 560 and the DNA marker SIUC-SAT185, excluding DNA marker 560. Taken together, the recombinant analyses suggested that the interval (∼14.3 kb) carrying DNA markers 570, 580 and 590 was underlying resistance to SCN at the *rhg1-a* locus in Forrest. Three genes were identified within the 14.3 kb interval; one codes for an armadillo/β-catenin-like repeat (*Glyma18g02570*) at the interval carrying marker 570; the second is an amino acid transporter (*AAT*, *Glyma18g02580*) at the interval carrying marker 580; the third is a soluble N-ethylmelaimide sensitive factor (NSF) attachment protein (*GmSNAP18*, *Glyma18g02590*) at the interval carrying marker 590. The complementary DNAs for each of the three identified genes were cloned and sequenced. Amino acid alignments of the predicted armadillo/β-catenin-like repeat and amino acid transporter protein sequences of Forrest and Essex revealed identical amino acid sequences between both the resistant and susceptible lines (Supplementary Fig. 1). A *GmSNAP18* genomic DNA sequence comparison of Forrest and PI 88788 with Essex identified six SNPs (C2447A, C2464G, G4203C, G4206C, G4206T and C4215A) and four InDels (−4211G, −4212G, −4213T and −4213G) within the exons (Fig. 1b), resulting in nine amino acid changes (Fig. 1c). Thus, *GmSNAP18* was deemed as the strongest candidate gene for conferring resistance to SCN at the *rhg1-a* locus.

### SNPs and InDels of the targeted *rhg1* genomic DNA segment

In total, 68,638, 68,986, 179,088 and 128,819 high-quality reads with an average coverage of about 22.5 × , 22.6 × , 58.5 × and 42.3 × were obtained from the RSE-Seq of the targeted 300 kb *rhg1* genomic DNA segment of Essex, Forrest, Peking and PI 88788, respectively. These reads were then mapped to the Williams 82 genomic sequence. Compared with the reference genomic sequence of Williams 82, 1,472 SNPs and InDels (1081 SNPs, 183 insertions and 208 deletions) were identified at the targeted 300 kb *rhg1* genomic DNA segment (Gm18: 1480001..1780000) of the soybean lines Essex, Forrest, Peking and PI 88788 (Table 1 and Supplementary Data 1), equating to 4.91 polymorphisms per kb of genomic DNA. Within the targeted 300 kb region, 632 SNPs, 109 insertions and 146 deletions were identified in Essex, whereas 835 (618 SNPs, 120 insertions and 97 deletions), 872 (649 SNPs, 123 insertions and 100 deletions) and 1,021 (736 SNPs, 120 insertions and 165 deletions) SNPs and InDels were identified in Forrest, Peking and PI 88788, respectively (Table 1 and Supplementary Data 1). This came to about 2.96, 2.78, 2.91 and 3.40 polymorphisms per kb of genomic DNA in Essex, Forrest, Peking and PI 88788, respectively.

**Figure 2:**
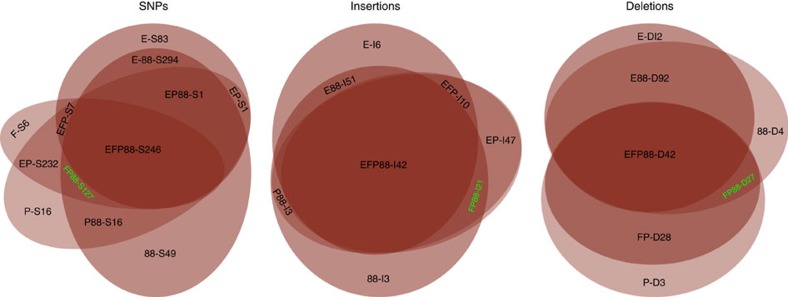
Analysis of the polymorphisms associated with the alleles underlying resistance to SCN. The results suggest that 127 SNPs (FP88-S127, green), 21 insertions (FP88-I21, green) and 27 deletions (FP88-D27, green) possessed by Forrest, Peking and PI88788, but not by Essex, are most possibly associated with the alleles underlying resistance to SCN. The number after S, I and D stands for the number of SNPs (S), insertions (I) or deletions (D), respectively. EFP88, EFP, EF88, EP88, FP88, EP, E88, FP, F88 and P88 stand for the overlapped SNPs, insertions or deletions of Essex (E), Forrest (F), Peking (P) and PI 88788 (88), respectively.

Among all of the polymorphisms called, there are many overlapping polymorphisms. As shown in Fig. 2, 246 SNPs (EFP88-S246), 42 insertions (EFP88-I42) and 42 deletions (EFP88-D42) are found in common among Essex, Forrest, Peking and PI 88788. In addition, 127 more SNPs (FP88-S127), 21 more insertions (FP88-I21) and 27 more deletions (FP88-D27) are in common among Forrest, Peking and PI 88788. Forrest and Peking uniquely retained 232 SNPs (FP-S232), 47 insertions (FP-I47) and 28 deletions (FP-D28). Peking and PI 88788 uniquely retained 19 SNPs (P88-S19) and 3 insertions (P88-I3), whereas PI 88788 uniquely retained 49 SNPs (88-S49), 3 insertions (88-I3) and 4 deletions (88-D4).

### Direct identification of the *rhg1* candidate gene *GmSNAP18*

Compared with susceptible soybeans, we identified 408 SNPs (FP88-S127, FP-S232 and 88-S49), 71 insertions (FP88-I21, FP-I47 and 88-I3) and 59 deletions (FP88-D27, FP-D28 and 88-D4) in the resistant Forrest, Peking and/or PI 88788 (Fig. 2 and Supplementary Data 1). Among them, 175 SNPs and InDels, including 127 SNPs (FP88-S127), 21 insertions (PF88-I21) and 27 deletions (PF88-D27), were considered to be associated with the alleles underlying resistance to SCN. Furthermore, 26 SNPs and InDels resulted in 25 amino acid changes (Supplementary Table 2 and Supplementary Data 1). However, when the genomic DNA sequences of the *GmSNAP18* from Forrest and PI 88788 were compared with those of the susceptible line Essex, only six SNPs (C2447A, C2464G, G4203C, G4206C, G4206T and C4215A) and four InDels (−4211G, −4212G, −4213T and −4213G) were mapped to the exons of gene *Glyma18g02590* (*GmSNAP18*). The identified polymorphisms resulted in nine amino acid changes (Supplementary Table 2). Thus, *GmSNAP18* was identified to be the most probable candidate gene conferring resistance to SCN at the *rhg1* locus, consistent with the results of map-based cloning (Fig. 1). The predicted *GmSNAP18* protein of Forrest and Peking is different from that of PI 88788. This is consistent with previous genetic results (termed as *rhg1-a* in Peking-type soybeans and *rhg1-b* in PI 88788-type soybeans)^4^.

### Multiple types of *GmSNAP18* at the *rhg1* locus

The RSE-Seq data indicated that multiple types of *GmSNAP18* existed within *rhg1* (Gm18: 1480001..1780000). In Forrest and Peking, only one type was identified within the whole segment between *Glyma18g02570* and *Glyma18g02610*, whereas PI 88788 was different. All types of *GmSNAP18* within the exons with or without amino acid changes, compared with the predicted *GmSNAP18* protein of Williams 82, are summarized in Table 2. PI 88788 showed two types of *GmSNAP18* (Type I, whose sequence is identical to that in the map-based cloning section mentioned above, and Type II, identical to the specific type of Williams 82 and Essex) at amino acid positions 203 and 285–288. These two types of *GmSNAP18* are the same as the two types of *GmSNAP18* in PI 88788 reported previously^7^,^9^. However, the third type of *GmSNAP18* in PI 88788 (deletion at position 203) reported previously^7^ was not detected within the 150 or more RSE-Seq reads at each position. The ratio of number of reads of Type I and Type II of the *GmSNAP18* in PI 88788 is about 8–9:1. Meanwhile, the sole type of *GmSNAP18* in both Forrest and Peking is different from the two types of *GmSNAP18* in PI 88788. In addition, *Glyma18g02610* also exhibited two types at the 5′-untranslated region in PI 88788: one is the same as that of Williams 82, whereas the other is a new type identical to that of Forrest and Peking.

### Haplotypes of the *GmSNAP18* predict SCN resistance

**Figure 3:**
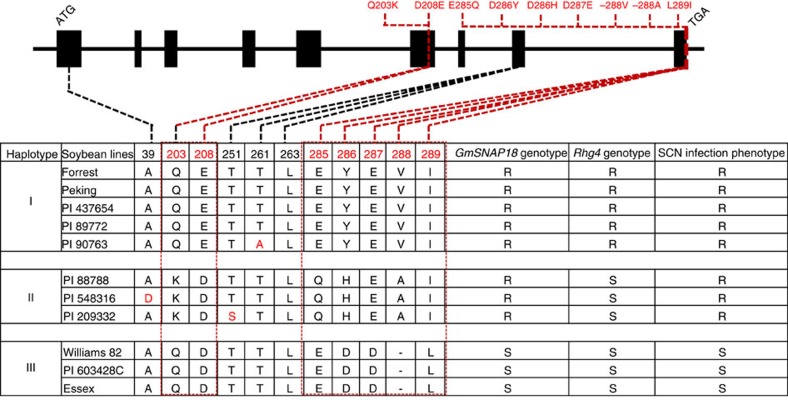
Haplotypes identified at *GmSNAP18* in 11 soybean lines. The *GmSNAP18* coding region for the 11 lines shown here was sequenced. The amino acid differences resulting from the nucleotide polymorphisms in the predicted protein sequences of *GmSNAP18* in 11 soybean lines are shown in the top and bottom panels where the number indicates the position of an amino acid in the predicted protein. The nucleotide polymorphisms and amino acid differences detected in the exons among Forrest, PI 88788, and Essex are in red boxes with dotted lines linking them. Haplotyping results from these 11 soybean lines clearly indicate three types of *GmSNAP18* haplotypes: two resistant types (Peking-Type I including Peking, Forrest, PI 437654, PI 89772 and PI 90763; and PI 88788-Type II including PI 88788, PI 548316 and PI 209332) and one susceptible Type III including Essex, Williams 82 and PI 603428C.

To establish a link between *GmSNAP18* alleles and soybean resistance to SCN, we scored 81 soybean lines (including PIs, landraces and elite cultivars, as described previously^6^) representing 95% of the sequence diversity of soybeans for their SCN female index (FI)^17^ and then determined their SNP-based *GmSNAP18* haplotype after genotyping them at the *rhg1* and *Rhg4* loci (Table 3). The genotyping data were clustered (Fig. 2 and Supplementary Table 1 of Liu *et al*.^6^) and 11 soybean lines were selected to fully sequence their *GmSNAP18* genes. As a result, 14 polymorphisms (10 SNPs and 4 InDels) at 13 positions were identified within the exons of *GmSNAP18* (Fig. 3). In total, three different *GmSNAP18* haplotypes were identified. Soybean lines with Haplotype I carry resistant alleles at both *GmSNAP18* and *Rhg4* (*GmSHMT08*), and are resistant to SCN (Table 3 and Liu *et al*.^6^). This includes soybean lines PI 548402 (Peking), Forrest, PI 90763, PI 437654 and PI 89772, all of which exhibit ‘Peking-type' resistance and require both *rhg1* and *Rhg4* (*GmSHMT08*). Soybean lines with Haplotype III carry the susceptible *GmSNAP18*, but vary for either the resistant or susceptible allele at the *Rhg4 GmSHMT08* (Table 3 and Liu *et al*.^6^). These lines are susceptible to SCN, regardless of the *Rhg4 GmSHMT08* genotype, and include soybean cultivars Essex, Williams 82 and PI 603428C. Soybean lines with Haplotype II carry the resistant allele at *GmSNAP18*, but vary for either the resistant or susceptible allele at the *Rhg4 GmSHMT08* (Table 3 and Liu *et al*.^6^). These lines are resistant to SCN and include PI 88788, PI 209332 and PI 548316 (*cv*. Cloud), all of which exhibit ‘PI 88788-type' resistance. In summary, the *GmSNAP18* haplotyping analysis is in agreement with previous SCN-resistance QTL reports and confirms the requirement of *rhg1* for the SCN resistance in both PI 88788 and Peking.

### Expression analyses of *GmSNAP18*

**Figure 4:**
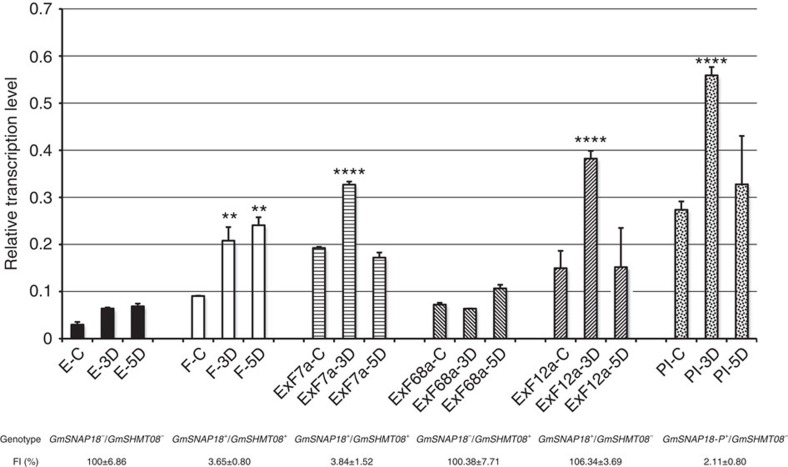
Quantitative RT–PCR analyses of *GmSNAP18* in the roots at different days after SCN infection and phenotype assays of soybean lines and ExF RILs with different genotypes of *GmSNAP18* and *GmSHMT08*. Roots without SCN infection were used as control. D, days after SCN infection; E, Essex; F, Forrest; PI, PI 88788. *GmSNAP18*^+^ and *GmSNAP18*^−^, and *GmSHMT08*^+^ and *GmSHMT08*^−^ represent Forrest and Essex *GmSNAP18*, and Forrest and Essex *GmSHMT08*, respectively; *GmSNAP18-P*^+^ represents PI 88788 *GmSNAP18*. Five replicates each line were performed, except for few lines with only three or four replicates due to one or two seeds that did not germinate. The error bar stands for the s.e.m. Asterisks indicate significant differences between samples as determined by *t*-test (****P<.0001 and **P<.01).

To gain more insight into the genetic responses of *GmSNAP18* to SCN, we analysed the expression of *GmSNAP18* in Essex, Forrest and PI 88788 during SCN infection through quantitative reverse transcriptase–PCR (qRT–PCR). The results (Fig. 4) show that the transcripts of *GmSNAP18* were induced in the resistant lines Forrest and PI 88788, whereas the susceptible line Essex showed very low transcription levels of *GmSNAP18* during SCN infection. Without SCN infection, *GmSNAP18* was expressed 2.1 times more in Forrest than in Essex and 8.3 times more in PI 88788 than in Essex. Under SCN infection, *GmSNAP18* transcripts were induced 2.3 and 2.5 times more in Forrest than in uninfected Forrest, and 2 and 1.1 times more in PI 88788 than in uninfected PI88788 at 3 and 5 days after SCN infection, respectively.

We successively analysed the *GmSNAP18* expression in the ExF RILs together with *GmSNAP18* and *GmSHMT08* genotyping and phenotyping (Fig. 4 and Supplementary Data 2). In this work, *GmSNAP18*^+^ and *GmSNAP18*^−^, and *GmSHMT08*^+^ and *GmSHMT08*^−^ represent Forrest and Essex *GmSNAP18*, and Forrest and Essex *GmSHMT08*, respectively; *GmSNAP18-P*^+^ represents PI 88788 *GmSNAP18*. The results demonstrate that *GmSNAP18* transcripts were induced in the ExF RILs carrying *GmSNAP18*^+^*/GmSHMT08*^+^ (ExF7) and *GmSNAP18*^+^*/GmSHMT08*^−^ (ExF12), whereas the ExF RILs carrying *GmSNAP18*^−^*/GmSHMT08*^+^ (ExF68) did not show significant changes in the expression of *GmSNAP18*, after SCN infection (3 days). ExF RILs carrying *GmSNAP18*^+^*/GmSHMT08*^+^ display resistance to SCN, whereas the ExF RILs carrying *GmSNAP18*^−^*/GmSHMT08*^+^ or *GmSNAP18*^+^*/GmSHMT08*^−^ do not show resistance to SCN (Fig. 4 and Supplementary Data 2). These results further support a role for *GmSNAP18* in resistance to SCN.

### Genetic complementation of Forrest *rhg1-a* resistance

**Figure 5:**
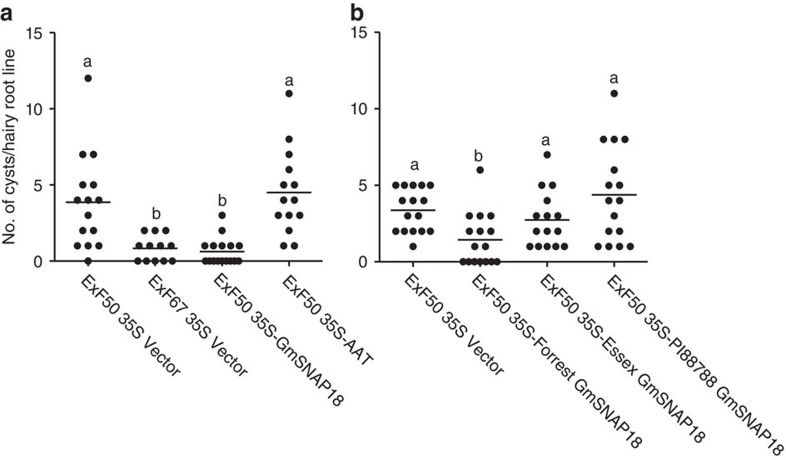
SCN infection of transgenic hairy roots of ExF50 expressing *GmSNAP18* or *AAT*. (**a**) SCN infection of RIL ExF50 transgenic hairy roots expressing Forrest *GmSNAP18* or *AAT*. Vector-transformed ExF50 (SCN-susceptible) and ExF67 (SCN-resistant) hairy roots were used as controls. Each dot represents the number of cysts on a single hairy root line. The number of dots corresponds to the number of independent hairy root lines used. Mean values significantly different from the control were determined using an unpaired *t*-test (*P*<0.0004) and are denoted by different letters. Similar results were obtained from at least five independent experiments with Forrest *GmSNAP18* and at least two independent experiments with *AAT*. Data from one representative experiment are shown. (**b**) SCN infection of RIL ExF50 transgenic hairy roots expressing *GmSNAP18* of Forrest, Essex or PI 88788. Vector-transformed ExF50 (SCN-susceptible) hairy roots were used as a control. Each dot represents the number of cysts on a single hairy root line. The number of dots corresponds to the number of independent hairy root lines used. Mean values significantly different from the control were determined using an unpaired *t*-test (*P*<0.0015) and are denoted by different letters. Similar results were obtained from at least three independent experiments. Data from one representative experiment are shown.

In Forrest, both *rhg1-a* and *Rhg4* alleles are required for resistance to SCN. VIGS silencing of Forrest *GmSNAP18* (*rhg1-a*) in resistant soybean RIL ExF67 carrying *GmSNAP18*^+^/*GmSHMT08*^+^ resulted in a strong cell death phenotype in the shoots, ultimately killing the plant (Supplementary Fig. 2). Therefore, to test the Forrest *GmSNAP18* for contribution to *rhg1-a* resistance, we used the SCN-susceptible RIL ExF50 carrying *GmSNAP18*^−^/*GmSHMT08*^+^, which has the resistant Forrest allele at the *Rhg4* locus and the susceptible Essex allele at the *rhg1* (*rhg1-s*) locus. As *GmSNAP18* occurs in multiple copies at this locus in the resistant soybean^7^,^8^,^9^, the construct was made to express the gene under the control of a CaMV 35S promoter *in planta*. Roots of RIL ExF67 transformed with the plasmid vector were used as a positive control for resistance. The transgenic hairy roots overexpressing Forrest *GmSNAP18* (*rhg1-a*) grew normally, but showed a significant reduction in SCN development compared with the control transgenic hairy roots in infection assays (Fig. 5a and Supplementary Figs 3a and 4). We also tested the amino acid transporter (*AAT*, *Glyma18g02580*, the predicted protein sequences between Forrest and Essex are identical (Supplementary Fig. 1) and did not observe a significant reduction in SCN development compared with the control transgenic hairy roots in infection assays (Fig. 5a and Supplementary Figs 3b and 4). The predicted protein sequences of *Glyma18g02570* of both Forrest and Essex are identical to that of Williams 82 (http://soybase.org) (Supplementary Fig. 1). *Glyma18g02570* did not contribute to SCN resistance^7^ and therefore was not tested in this study. These data suggest that the *GmSNAP18* gene at the *rhg1-a* locus is sufficient to confer SCN resistance in Forrest. We also expressed the *GmSNAP18* genes from two other soybean lines: Essex (*rhg1-s*) and PI 88788 (*rhg1-b*). Expression of the *GmSNAP18* gene from either Essex or PI 88788 in RIL ExF50 did not result in a significant reduction in SCN development compared with the control (Fig. 5b and Supplementary Fig. 4), demonstrating the specificity of Forrest *GmSNAP18* in conferring Peking-type soybean resistance to SCN.

### Modelling of GmSNAP18

**Figure 6:**
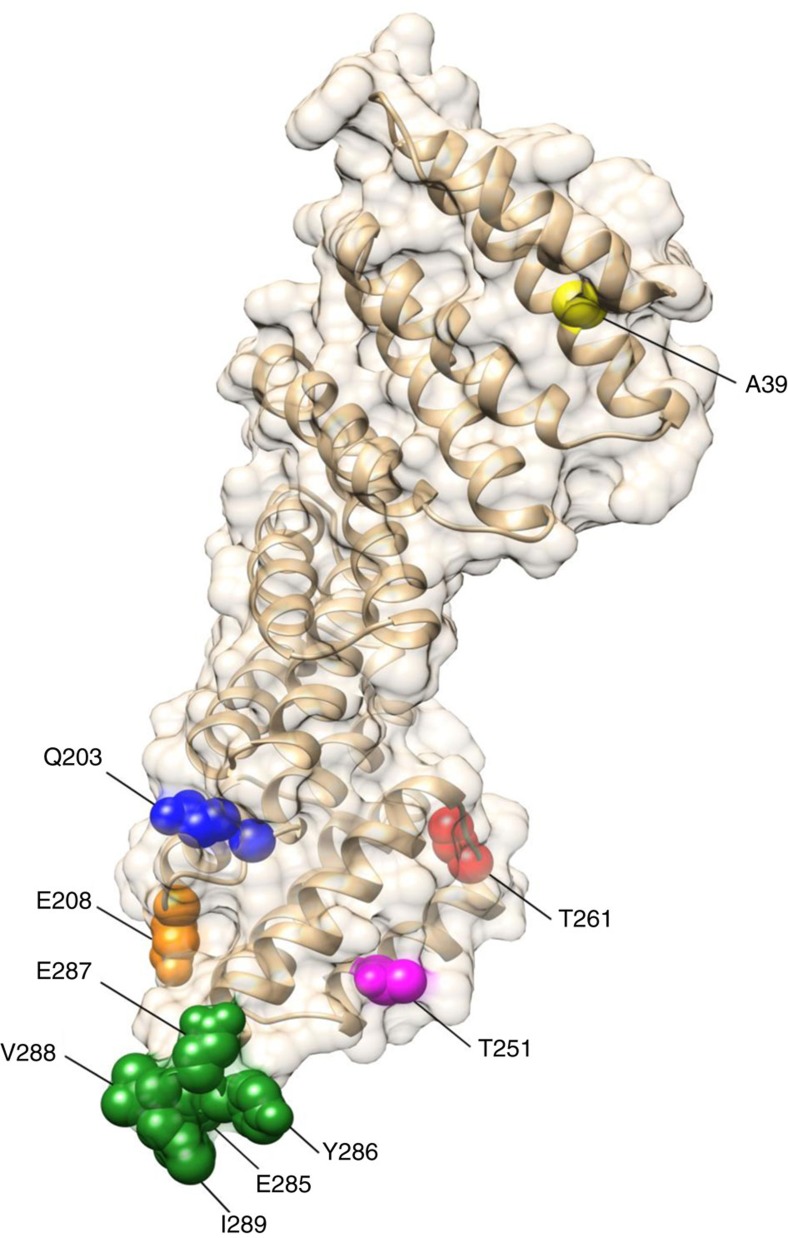
Homology model of the *GmSNAP18* from Forrest with mapped residues of identified haplotypes. The C-terminal residues 285-289 (E285, Y286, E287, V288 and I289) vary among all the three *GmSNAP18* types; the residue E208 varies in *GmSNAP18* Types II and III; the residue Q203 varies in *GmSNAP18* Type II; the residue T261 varies only in PI 90763 (from Type I); the residue T251 varies only in PI 209332 (from Type II); and the residue A39 varies only in PI 548316 (from Type II).

To understand the locations of the different identified haplotypes of *GmSNAP18* and how they may confer SCN resistance, the Forrest *GmSNAP18* was modelled and then each haplotype of the other soybean lines (Fig. 3) was mapped. The modelling showed that most of the identified differences were at the carboxy-terminus of the GmSNAP18. These haplotypes not only varied between PI 88788-type and Peking-type resistant lines, but also between the resistant and susceptible lines (Fig. 6). Through structural analyses, variations in the *GmSNAP18* C-terminal residues 285–289 (E285, Y286, E287, V288 and I289) may play a role in establishing protein–protein interactions or modulating vesicular exocytosis^18^. It has been reported that the C-terminus of soluble N-ethylmaleimide sensitive fusion attachment proteins (SNAPs; last 25 residues) determines its functionality in vesicle trafficking and localization^19^. The Q203K haplotype in PI 88788-type soybeans (Fig. 3) has an altered charge of the immediate environment from an uncharged glutamine to a positively charged lysine. The T261A variation only occurring in PI 90763 (Fig. 3) shows a polarity shift from a polar threonine in all other soybean lines to a non-polar alanine, which probably enables PI 90763 to acquire new resistance to certain SCN Hg Types. Similarly, the T251S variation in PI 209332 and A39D in PI 548316 may play a role in nematode effector recognition, as this ability differs from its closest soybean haplotypes (Fig. 3).

## Discussion

In this study, *GmSNAP18* was identified as the *rhg1-a* gene conferring resistance to SCN using an integrated set of genomic and genetic approaches in combination with genetic complementation analyses. A high-density genetic map of a 370 kb chromosomal segment carrying the Forrest *rhg1-a* locus was developed using three RIL populations (Fig. 1a). Three recombination events (ExF3126, ExF4361 and WxF6034) narrowed the *rhg1-a* gene candidate to a region containing three genes, *Glyma18g02570*, *Glyma18g02580* and *Glyma18g02590* (*GmSNAP18*) (Fig. 1a). Similar to Peking^7^, three tandem copies of the *Glyma18g02570*, *Glyma18g02580* and *Glyma18g02590* are present within the *rhg1*-a locus in the cultivar Forrest. The recombinant WxF6034 carried the Forrest allele for *Glyma18g02610*, but was susceptible to SCN, indicating that the *Glyma18g02610* gene is not contributing to the SCN resistance in the Forrest background. Previously, the leucine-rich repeat receptor-like kinase (*LRR-RLK*) genes at the *rhg1* and *Rhg4* loci were claimed as the resistance genes^20^,^21^,^22^,^23^,^24^. However, Melito *et al*.^25^ and Liu *et al*.^26^ demonstrated that the *LRR-RLK* genes at the *rhg1* and *Rhg4* loci did not confer SCN resistance. In the present work, the *rhg1-a* genetic map of susceptible RIL WxF6034 clearly showed that WxF6034 carried the Forrest genotype at the TMD1 marker and the flanking region carrying the *LRR-RLK* gene (Fig. 1a), excluding the *rhg1-a LRR-RLK* candidate resistance gene in support of earlier work.

To further confirm the reliability of the constructed genetic map, RSE-Seq was used to study the SNPs and InDels among resistant and susceptible genotypes to pinpoint candidate genes for resistance to SCN at the *rhg1* locus. RSE has been successfully applied to yeast, human and zebrafish^11^,^12^,^13^,^14^,^15^, but has not yet been applied to plants. Here we show that RSE-Seq of a targeted 300 kb genomic DNA segment (Gm18: 1480001..1780000) of contrasting chromosomal regions underlying resistance was effective in the direct identification of a candidate *rhg1* SCN resistance gene (Table 1 and Fig. 2). We postulated that the SNPs and InDels at *rhg1* most likely to be associated with the alleles underlying resistance to SCN would be shared by Forrest, Peking, and/or PI 88788, but not present in the genomic DNA of susceptible line Essex at the *rhg1* locus. Furthermore, those with changes within exons that resulted in amino acid changes would be considered high priority candidates. Compared with the genomic sequence of Williams 82 at that targeted 300 kb *rhg1* genomic DNA segment, the sequence analysis of Forrest, Peking and PI 88788 resulted in the identification of 835, 872, and 1021 SNPs and InDels, respectively. In contrast, Essex showed 886 SNPs and InDels (Table 1 and Supplementary Data 1), of which only 26 SNPs and InDels were potentially associated with the alleles underlying resistance to SCN (Supplementary Table 2). Furthermore, of those 26 SNPs and InDels, 10 were specific to Forrest, Peking, and PI 88788, and all were located within the exons of one gene *Glyma18g02590* (*GmSNAP18*). This resulted in nine amino acid changes to the predicted *GmSNAP18* protein (Supplementary Table 2). The other SNPs and InDels identified did not result in any amino acid changes. Thus, consistent with the genetic mapping results, *GmSNAP18* was identified as the *rhg1* candidate gene probably conferring resistance to SCN. The results obtained in this study suggest that the RSE method is a powerful tool for the preparation of specific genomic regions for next-generation sequencing (NGS). However, the design of specific primers is critical when working with organisms possessing repetitive genome sequences. For instance, the soybean genome is about 1.115 Gb in size^27^, but 40–60% of the soybean genome is repetitive sequence and heterochromatic^28^,^29^,^30^. To enrich the specific region (Gm18: 1480001..1780000), one primer was designed approximately every 5 kb for a total of 60 primers spanning the targeted genomic DNA region. Specific primers ensured that other homeologous genomic DNA segments (that is, chromosome 11) were not enriched to avoid biased sequence reads which could have complicated the sequence data analyses.

The genetic complementation analyses of *GmSNAP18* clearly showed that the transgenic ExF50 (*GmSNAP18*^−^*/GmSHMT08*^*+*^) gained resistance to SCN after being complemented with the Forrest *GmSNAP18*, but not when complemented with Forrest *AAT* (Fig. 5a and Supplementary Fig. 4), indicating that the Forrest *GmSNAP18* is the gene at the *rhg1-a* locus that confers resistance to SCN. In addition, the PI 88788 *GmSNAP18* (*rhg1-b*) or Essex *GmSNAP18* (*rhg1-s*) could not restore resistance to ExF50 (Fig. 5b and Supplementary Fig. 4), showing that the only form of *GmSNAP18* that could complement the ExF50 RIL is the Forrest-type *GmSNAP18* (*rhg1-a*). These data suggest that the resistant form of the PI 88788 *GmSNAP18* is unable to communicate with the Forrest *GmSHMT08* (*Rhg4*), to confer resistance to SCN. Our genetic mapping results did not include *Glyma18g02610* among the *rhg1-a* candidate genes (Fig. 1a). Thus, the complementation results indicate that the SCN resistance at the *rhg1-a* locus in Forrest is triggered by *GmSNAP18* alone, contrasting with the *rhg1-b* in PI 88788, whose resistance is due to the high copy number and concerted activity of three genes^7^.

Forrest, Peking and PI 88788 require the presence of either the resistant *rhg1-a* or *rhg1-b* allele to exhibit resistance to SCN^2^,^3^,^6^,^7^. In addition, the resistance of Forrest and Peking to SCN requires the *Rhg4* gene^6^. The resistance reaction of Peking-type soybeans to nematodes is quick and forceful leading to a rapid degeneration of the syncytium, whereas PI 88788-type soybeans react to nematodes comparatively slower^31^. SCN populations that have adapted to reproduce on soybeans with either Peking-type or PI 88788-type resistance have been reported^32^. According to the different haplotypes of *GmSNAP18* identified from a collection of 81 soybean lines, Peking, Forrest, PI 90763, PI 437654 and PI 89772 were classified as Peking-type soybeans; PI 88788, PI 209332 and PI 548316 were classified as PI 88788-type soybeans; and Essex, Williams 82 and PI 603428C were classified as SCN-susceptible-type soybeans (Table 3 and Fig. 3). All are in agreement with the previous genetic results, showing that the SCN resistance of Peking-type soybeans such as Forrest requires both *rhg1-a* and *Rhg4* (*GmSHMT08*)^2^,^3^,^6^. The genomic and cDNA sequences of the *GmSNAP18* conferring SCN resistance were different between Peking-type soybeans and PI 88788-type soybeans (Fig. 1b), consistent with the different *rhg1* loci identified in both lines (*rhg1-a* and *rhg1-b*)^4^.

SNAPs are highly conserved proteins and belong to the tetratricopeptide repeat containing protein family. Tetratricopeptide repeat proteins are known to be involved in vesicular trafficking, cytokinesis and plasma membrane repair and stability^33^,^34^,^35^,^36^,^37^. SNAP proteins have been described as the member of the SNARE complex involved in many pathogen resistance pathways. It has been reported that SNAP, together with syntaxins SYP121 and SYP132, contributes to the resistance to fungi and bacteria^38^,^39^. The major amino acid changes of *GmSNAP18* between susceptible, Peking-type and PI 88788-type soybean lines were mostly mapped in the C-terminus of the protein (Fig. 6), which we classified into different haplotypes (Fig. 3). Considering that the C-terminus of SNAP proteins have been found to control vesicular trafficking^19^, the haplotype differences among susceptible, Peking-type and PI 88788-type soybeans may alter the destination of a GmSNAP18-guided vesicle. In addition to the two major resistant haplotypes (Peking and PI 88788), there are also the PI 90763 (T261A), PI 548316 (A39D), and PI 209332 (T251S) haplotypes. SNAP proteins have also been found to have their activity altered by phosphorylation. As most kinases act on serine or threonine, the T261A and T251S may have altered phosphorylation capabilities. Various α-SNAP haplotypes may also play a role in nematode effector recognition^40^ or in establishing protein–protein interactions^41^,^42^. Thus, the differences in *GmSNAP18* among soybean genotypes could determine the type of interactions between the nematode and the soybean host.

Taken together, our results support a model wherein *GmSNAP18* is a major factor mediating the different types of soybean resistance to SCN. Our findings suggest that the Peking-type *GmSNAP18* is performing a different role in SCN resistance than PI 88788-type *GmSNAP18*. This is an example of a pathogen resistance gene that has evolved to underly two types of resistance, yet ensure the same function within a single plant species. Now that the major genes for resistance to SCN have been discovered, the molecular and biochemical mechanisms of soybean resistance to SCN can be explored in detail.

## Methods

### Nematode and plant materials

The inbred SCN population PA3 (Hg Type 0) used in this study was mass selected on soybean *cv*. Williams 82 according to standard procedures^32^ at the University of Missouri. The soybean cultivars Forrest^43^, Peking and PI88788 are resistant to SCN PA3. The soybean cultivars Essex^44^ and Williams 82 (ref. 45) are susceptible to SCN PA3. The three RIL populations (crosses between Forrest and Essex (ExF) or Williams 82 (FxW or WxF), 3,913 RILs in total) were bred by Iowa State University and phenotyped for SCN resistance by the University of Missouri using established methodology^46^. The collection of soybean lines used in this study was obtained from the United States Department of Agriculture (USDA) Soybean Germplasm Collection, University of Illinois.

### Extraction of soybean genomic DNA

The soybean seeds were planted in the greenhouse at Southern Illinois University, germinating and growing at 25–30 °C. One top young trifoliate leaf of a seedling from each soybean line was collected for the extraction of genomic DNA about 2 weeks after germination. The genomic DNA extraction was performed using a Qiagen DNeasy Plant Mini Kit or a Qiagen DNeasy 96 Plant Kit (Qiagen Sciences, USA) per the manufacturer's manual instructions. The DNA extracted was quantified to 100 ng μl^−1^ for RSE-Seq, genetic mapping, haplotyping and TILLING mutation screening.

### Map-based cloning of the *rhg1-a* gene

Three genetic populations segregating for resistance to SCN PA3 (Hg Type 0) were used for mapping. These included an F_5:6_ RIL population from a cross between Forrest and Essex (98 individuals) and two large F_5:6_ RIL populations generated from crosses between Forrest and either Essex (1,755 lines) or Williams 82 (2,060 lines) to enrich the chromosomal interval carrying the *rhg1* gene recombinants.

As Forrest resistance to SCN requires both the *rhg1-a* and *Rhg4* genes^3^, genotyping was conducted using DNA markers flanking both loci to detect informative recombinants at the *rhg1* locus (Supplementary Table 1). The simple sequence repeat (SSR) markers Sat_210, Satt309 and SIUC-SAT143 were used to identify chromosomal breakpoints at the *rhg1-a* locus. PCR amplifications were performed using DNA from individuals from each of the three genetic populations. Cycling parameters were as follows: 35 cycles of 94 °C for 30 s, 50 °C for 30 s and 72 °C for 30 s with 7 min of extension at 72 °C. The PCR products were separated on 3–4% metaphor agarose gels. The identified recombinants were subjected to a second screening by using the EcoTILLING marker SHMT^6^ and the simple sequence repeat (SSR) marker Sat_162 to identify the *Rhg4* genotype of each recombinant.

To enrich the chromosomal regions carrying the *rhg1-a* locus with DNA markers, the GenBank published sequence AX196295 spanning the region and the Williams 82 reference sequence on http://soybase.org, spanning this region were used to design PCR primers every 5–10 kb of the 370 kb carrying the *rhg1* locus (Supplementary Table 1). DNA from Forrest, Essex and Williams 82 was tested with each primer, using a modified EcoTILLING protocol, to find and map polymorphic sequences at the *rhg1* locus^6^,^26^,^47^. The identified SNP and InDel DNA markers were integrated into the informative recombinants to identify chromosomal breakpoints and the interval that carried the *rhg1-a* gene.

### Isolation of the genomic and cDNA sequences of *GmSNAP18*

The genomic DNA and cDNA of *GmSNAP18* in Forrest, Essex and PI 88788 were cloned and sequenced. Genomic DNA was isolated from young leaves using the DNeasy Plant Mini Kit (Qiagen Sciences). Total RNA was isolated from roots using the RNeasy Plant Mini Kit (Qiagen Sciences) and cDNA was synthesized using a cDNA synthesis kit (Invitrogen, USA). PCR primers based on the Forrest and Essex genomic DNA sequences were used to amplify the corresponding cDNA sequences (Supplementary Table 1).

### Enrichment of one targeted 300 kb *rhg1* genomic DNA segment

Using Williams 82 genomic sequence as the reference sequence, a specific oligonucleotide primer was designed about each 5 kb between Gm18: 1480001 and 1780000 for the RSE of one targeted 300 kb *rhg1* genomic DNA segment (Supplementary Table 3). RSE was performed using the genomic DNA of Essex, Forrest, Peking and PI 88788 as described by Gabriel *et al*.^11^ and Dapprich *et al*.^12^, by Generation Biotech (Lawrenceville, NJ, USA). Briefly, capture primers designed were designed to hybridize to targeted areas of the genome by exploiting sequence elements that are unique to the region of interest. The bound oligos are extended with biotinylated nucleotides to label the targeted DNA segments. Streptavidin-coated magnetic microparticles are then added to the reaction mix to isolate the targeted DNA, along with its flanking regions. The 30 μl RSE reaction mix consisted of a premixed set of targeting primers combined with 600 ng of genomic DNA. The genomic DNA was denatured and an automated capture was performed, followed by washing and elution in preloaded reagent cartridges. After RSE, the enriched DNA from each sample was removed from the microparticles by heating the solution at 80 ^o^C for 15 min to disrupt the biotin–streptavidin complex^12^. The microparticles were magnetically collected and the elute containing the enriched regional genomic DNA was retained for theNGS.

### NGS of the targeted 300 kb *rhg1* genomic DNA segment

The NGS of the targeted 300 kb *rhg1* genomic DNA segment of the soybean lines Essex, Forrest, Peking and PI 88788 was carried out by GenXPro GmbH in Frankfurt, Germany. Briefly, the enriched samples RSEed in the last step were amplified with an Illustra GenomiPhi V2 DNA amplification kit (GE Healthcare) according to the manufacturer's protocol. Residual primers and dNTPs were deactivated with ExoSAP-IT as per the manufacturer's protocol. For each sample, approximately 2 μg of enriched, amplified genomic DNA was used as input for the preparation of the sequencing library. The library was prepared for sequencing using the Illumina Genomic DNA Sample Prep Kit. Sequencing was performed using an Illumina Hiseq2000.

### Reads mapping and calling of SNPs and InDels

After the reads were analysed and filtered following sequencing, the high-quality reads were mapped with novoalign version 2.07.14 to the Williams 82 genomic sequence, resulting in a BAM file for each soybean line with the SAMTools^48^. The sequences were analysed with a Tablet viewer^49^ and an IGV viewer^50^ for the building of consensus sequences, and the manual calling of SNPs and InDels using the Williams 82 genomic sequence as a reference sequence.

### Haplotyping of *GmSNAP18* in soybean lines

A total of 81 soybean lines (PIs, landraces and elite cultivars), representing 95% of the genetic variability of soybeans^17^, were scored for their SCN FI. Lines were classified resistant (R) to SCN if the FI was ≤10% and susceptible (S) if the FI was >10%. Soybean lines were genotyped at the *rhg1* locus by using the DNA markers 560, 570 and Satt309. They were also genotyped at the *Rhg4* locus, using the DNA marker Sat_162 and by the sequencing of *GmSHMT08* (ref. 6). The coding region of *GmSNAP18* was sequenced for 11 lines. Common SNPs and InDels were identified and used to determine the different *GmSNAP18* haplotypes.

### Quantitative RT–PCR of the *GmSNAP18* gene

Soybean seedlings from the susceptible line Essex, the resistant lines Forrest and PI 88788, and two RILs ExF7 (*GmSNAP18*^+^*/GmSHMT08*^+^) and ExF68 (*GmSNAP18*^−^*/GmSHMT08*^+^) were grown in autoclaved sandy soil in the growth chamber for 1 week and then infected with infective eggs from the PA3 population. Total RNA was isolated from root samples 3 and 5 days after SCN infection using Qiagen RNeasy Plant Mini Kit (Qiagen Sciences). Total RNA was DNase treated and purified using a Turbo DNA-free Kit (Life Technologies, USA). RNA was quantified using NanoDrop 1000 (V3.7), then a total of 400 ng of treated RNA was used to generate cDNA using the cDNA synthesis kit (Life Technologies) with random hexamers and 2 μl of cDNA was used for the *GmSNAP18* quantitative PCR with the specific primers (Supplementary Table 1), using the Power SYBR Green PCR Master Mix kit (Life Technologies). Gene transcription from three individual biological replicates was used for quantification, then normalized by the deltadelta *C*_q_ method using Ubiquitin as a reference gene (Δ*C*_q_=*C*_q(TAR)_–*C*_q(REF)_). *GmSNAP18* expression was exponentially converted using the formula: Δ*C*_q_ Expression=2^−Δ*C*q^.

### VIGS infection analyses

BPMV VIGS vectors pBPMV IA-R1M and pBPMV-IA-V1 were used in this study^51^. Briefly, a 302 bp fragment (spanning base pairs 365–667) of *GmSNAP18* (Glyma18g02590) cDNA sequence (GenBank accession number: KX147332) was amplified from soybean *cv*. Forrest root cDNA by RT–PCR, using primers listed in Supplementary Table 1. PCR products were digested with BamH1 and ligated into pBPMV-IA-V1, then digested with the same enzyme to generate pBPMV-SNAP18-AS (BPMV-SNAP18-AS) with the insert in the antisense orientation. Gold particles coated with pBPMV-IA-R1M and BPMV-SNAP18-AS were co-bombarded into soybean leaf tissue as described previously^52^. For vector control, pBPMV-R1M and pBPMV-GFP-AS were used. At 3–4 weeks after inoculation, trifoliate leaves showing virus symptoms were collected, lyophilized and stored at −20 °C to be used as virus inoculum for plants. The SCN-resistant RIL ExF67 (*GmSNAP18*^+^/*GmSHMT08*^+^) seedlings were inoculated with virus carrying pBPMV-SNAP18-AS or a vector at 7–9 days post germination and the plants were maintained at 20 °C, 16 h light/8 h dark for 30 days post virus infection. The experiment was repeated twice with similar results.

### Candidate gene complementation experiments

Based on the aforementioned mapping data, the candidate gene *GmSNAP18* and a putative amino acid transporter (*AAT*) were selected for complementation experiments. For this, Forrest *GmSNAP18* and Forrest *AAT* were subcloned into a 35S pAKK gateway vector by recombination cloning. Briefly, the 873 bp open reading frame of *GmSNAP18* or the 1311, bp open reading frame of *AAT* were PCR amplified from Forrest root cDNA using gateway primers (Supplementary Table 1). They were cloned into pDONR/Zeo and subsequently moved into the gateway vector pAKK downstream of CaMV 35S promoter. Transgenic hairy roots were generated from the RIL ExF50, which carries the Forrest *Rhg4* (resistant allele) and Essex *rhg1* (*rhg1-s*, susceptible allele) as described previously^53^. Transgenic hairy roots of vector transformed RIL ExF67 (ref. 6) were used as an SCN-resistant control. The infection experiments were done in square Petri plates as described previously^6^. In all experiments, at least 12 independent transgenic hairy root lines were used per treatment. The experiments with *GmSNAP18* were repeated at least five times and those with *AAT* were repeated twice. For experiments with *GmSNAP18* of Essex (*rhg1-s*) or PI 88788 (*rhg1-b*), the constructs were made as described above, using *GmSNAP18* cloned from the root cDNA of these lines, respectively. Three biological replicates of transgenic root generation and infection assays were conducted as described above. The results from infection assays were plotted and analyzed for statistical significance by an unpaired *t*-test using GraphPad PRISM software.

### Modelling GmSNAP18

Homology modelling of a putative *GmSNAP18* protein structure was conducted with Deepview and Swiss Model Workspace software^54^, using the predicted *GmSNAP18* protein sequence from Forrest and an available α-SNAP crystal structure from *Rattus norvegicus* as a template; PDB accession is 3J96 chain G^55^,^56^,^57^. Residues 6–284 were modelled against this template with a sequence identity of 39%. The remaining residues were appended on the structure with Deepview, followed by an energy minimization using the ModRefiner server^58^. Haplotype mapping and visualizations were performed using the UCSF Chimera package^59^.

### Statistical analysis

All the presented qRT–PCR results were performed with the analysis of variance by Student's *t*-test means comparison using JMP Pro V12 software.

### Data availability

The genomic DNA and cDNA sequences of *GmSNAP18* in Forrest, Essex and PI 88788 have been deposited in NCBI GenBank with the accession number of KX147329, KX147331, KX147330, KX147332, KX147333 and KX147334, respectively. The sequences of the 300 kb *rhg1* segment of four soybean lines have been deposited in NCBI Sequence Read Archive with the study number of SRP090423. The authors declare that all other data supporting the findings of this study are available upon request.

## Additional information

**How to cite this article:** Liu, S. *et al*. The soybean *GmSNAP18* gene underlies two types of resistance to soybean cyst nematode. *Nat. Commun.* **8**, 14822 doi: 10.1038/ncomms14822 (2017).

**Publisher's note:** Springer Nature remains neutral with regard to jurisdictional claims in published maps and institutional affiliations.

## Supplementary Material

Supplementary Information Supplementary Figures, Supplementary Tables and Supplementary References

Supplementary Data 1 The detailed information of polymorphisms detected at the 300 kb region of Gm18 in 4 SCN-susceptible or -resistant soybean lines. Totally, 1472 SNPs (S), Insertions (I) and deletions (D) were detected including 1081 SNPs, 183 insertions and 208 deletions.

Supplementary Data 2 The SCN-infection phenotype of different genotypes of ExF RILs and their parents.

## Figures and Tables

**Table 1:** The polymorphisms detected at the targeted 300 kb region of Gm18 in four SCN-susceptible or -resistant soybean lines by RSE-Seq.

	**Essex**	**Forrest**	**Peking**	**PI 88788**	**Total**
SNPs	632	618	649	736	1,081
Insertions	109	120	123	120	183
Deletions	146	97	100	165	208
Total	887	835	872	1,021	1,472

RSE-Seq, region-specific extraction sequencing; SCN, soybean cyst nematode; SNP, single-nucleotide polymorphism.

**Table 2:** Multiple types of *GmSNAP18* of four SCN-susceptible or -resistant soybean lines identified by RSE-Seq compared with Williams 82

	Type	Reads no.	203	208	Type	Reads no.	285	286	287	288
**Williams 82**			**CAA Q**	**GAC D**			**GAG E**	**GAT D**	**GAT D**	**CTT L**
Essex	I	49	**CAA Q**	**GAC D**	I	56	**GAG E**	**GAT D**	**GAT D**	**CTT L**
Forrest	I	76	CAA Q	GAG E	I	92	GAG E	TAT Y	GAGGTT E V	ATT I
Peking	I	198	CAA Q	GAG E	I	152	GAG E	TAT Y	GAGGTT E V	ATT I
PI88788	I	163	AAA K	GAC D	I	150	CAG Q	CAT H	GAGGCT E A	ATT I
	II	19	**CAA Q**	**GAC D**	II	15	**GAG E**	**GAT D**	**GAT D**	**CTT L**

RSE-Seq, region-specific extraction sequencing; SCN, soybean cyst nematode.

**Table 3:** Genetic mapping of *rhg1* and *Rhg4* loci of 11 soybean lines using 4 DNA markers for *rhg1* locus and 1 DNA marker with *GmSHMT08* sequencing for *Rhg4* locus.

**Soybean lines**	**SCN infection phenotype**	***rhg1 Locus***				***Rhg4 locus***	
		**560**	**570**	**590**	**Satt309**	***GmSHMT08***	**Sat_162**
Forrest	R	R	R	R	R	R	R
Peking	R	R	R	R	R	R	R
PI 437654	R	R	R	R	R	R	R
PI 89772	R	R	R	R	R	R	R
PI 90763	R	R	R	R	R	R	R
							
PI 88788	R	R	R	R	S	S	R
PI 548316	R	R	R	R	S	S	R
PI 209332	R	R	R	R	S	S	S
							
Essex	S	S	S	S	S	S	S
Williams 82	S	S	S	S	S	S	S
PI 603428C	S	S	S	S	R	S	R

FI, female index; R, resistant; S, susceptible; SCN, soybean cyst nematode.The Forrest genotype is classified as the R genotype and the Essex genotype is the S genotype. Lines are classified R to SCN if FI ≤10% and S if FI >10%.
