# Supplementary material for: The soybean GmSNAP18 gene underlies two types of resistance to soybean cyst nematode
Source: Nat Commun. 2017 Mar 27;8:14822. doi: 10.1038/ncomms14822 (PMC5378975; doi:10.1038/ncomms14822)
Supplement: Supplementary Information — Supplementary Figures, Supplementary Tables and Supplementary References [file ncomms14822-s1.pdf]

a

```
Forrest        MRALAAQFSNYLCRRKVGVNLRSRNFSSYNSKDELTIEEEAERKVGWLLKTIFFVTAGVA
Essex          MRALAAQFSNYLCRRKVGVNLRSRNFSSYNSKDELTIEEEAERKVGWLLKTIFFVTAGVA
               ************************************************************

Forrest        GYHFFPYMGENLMQQSVSLLRVKDPLFKRMGASRLARFAVDDERRKKIVEMGGAQELLNM
Essex          GYHFFPYMGENLMQQSVSLLRVKDPLFKRMGASRLARFAVDDERRKKIVEMGGAQELLNM
               ************************************************************

Forrest        LSTAKDDRTRKEALHALDALSQSDEALASLHHAGAISVIRSAPNSLEDAEVEGFKLSLMK
Essex          LSTAKDDRTRKEALHALDALSQSDEALASLHHAGAISVIRSAPNSLEDAEVEGFKLSLMK
               ************************************************************

Forrest        RFQDLRYDVPS
Essex          RFQDLRYDVPS
               ***********
```

b

```
Forrest        MSPAAGVSVPLLGDSKGTPPPASVPGAVFNVATSIVGAGIMSIPAIMKVLGVVPAFAMIL
Essex          MSPAAGVSVPLLGDSKGTPPPASVPGAVFNVATSIVGAGIMSIPAIMKVLGVVPAFAMIL
               ************************************************************

Forrest        VVAVLAELSVDFLMRFTHSGETTTYAGVMREAFGSGGALAAQVCVIITNVGGLILYLIII
Essex          VVAVLAELSVDFLMRFTHSGETTTYAGVMREAFGSGGALAAQVCVIITNVGGLILYLIII
               ************************************************************

Forrest        GDVLSGKQNGGEVHLGILQQWFGIHWWNSREFALLFTLVFVMLPLVLYKRVESLKYSSAV
Essex          GDVLSGKQNGGEVHLGILQQWFGIHWWNSREFALLFTLVFVMLPLVLYKRVESLKYSSAV
               ************************************************************

Forrest        STLLAVAFVGICCGLAITALVQGKTQTPRLFPRLDYQTSFFDLFTAVPVVVTAFTFHFNV
Essex          STLLAVAFVGICCGLAITALVQGKTQTPRLFPRLDYQTSFFDLFTAVPVVVTAFTFHFNV
               ************************************************************

Forrest        HPIGFELAKASQMTTAVRLALLLCAVIYLAIGLFGYMLFGDSTQSDILINFDQNAGSAVG
Essex          HPIGFELAKASQMTTAVRLALLLCAVIYLAIGLFGYMLFGDSTQSDILINFDQNAGSAVG
               ************************************************************

Forrest        SLLNSLVRVSYALHIMLVFPLLNFSLRTNIDEVLFPKKPMLATDNKRFMILTLVLLVFSY
Essex          SLLNSLVRVSYALHIMLVFPLLNFSLRTNIDEVLFPKKPMLATDNKRFMILTLVLLVFSY
               ************************************************************

Forrest        LAAIAIPDIWYFFQFLGSSSAVCLAFIFPGSIVLRDVKGISTRRDKIIALIMIILAVVTS
Essex          LAAIAIPDIWYFFQFLGSSSAVCLAFIFPGSIVLRDVKGISTRRDKIIALIMIILAVVTS
               ************************************************************

Forrest        VLAISTNIYNAFSSKS
Essex          VLAISTNIYNAFSSKS
               ****************
```

Supplementary Figure 1. **The predicted protein sequences of Glyma18g02570 and Glyma18g02580 in Forrest and Essex.** (**a**) Alignment of the predicted protein sequences of Glyma18g02570 in Forrest and Essex; (**b**) Alignment of the predicted protein sequences of Glyma18g02580 in Forrest and Essex.

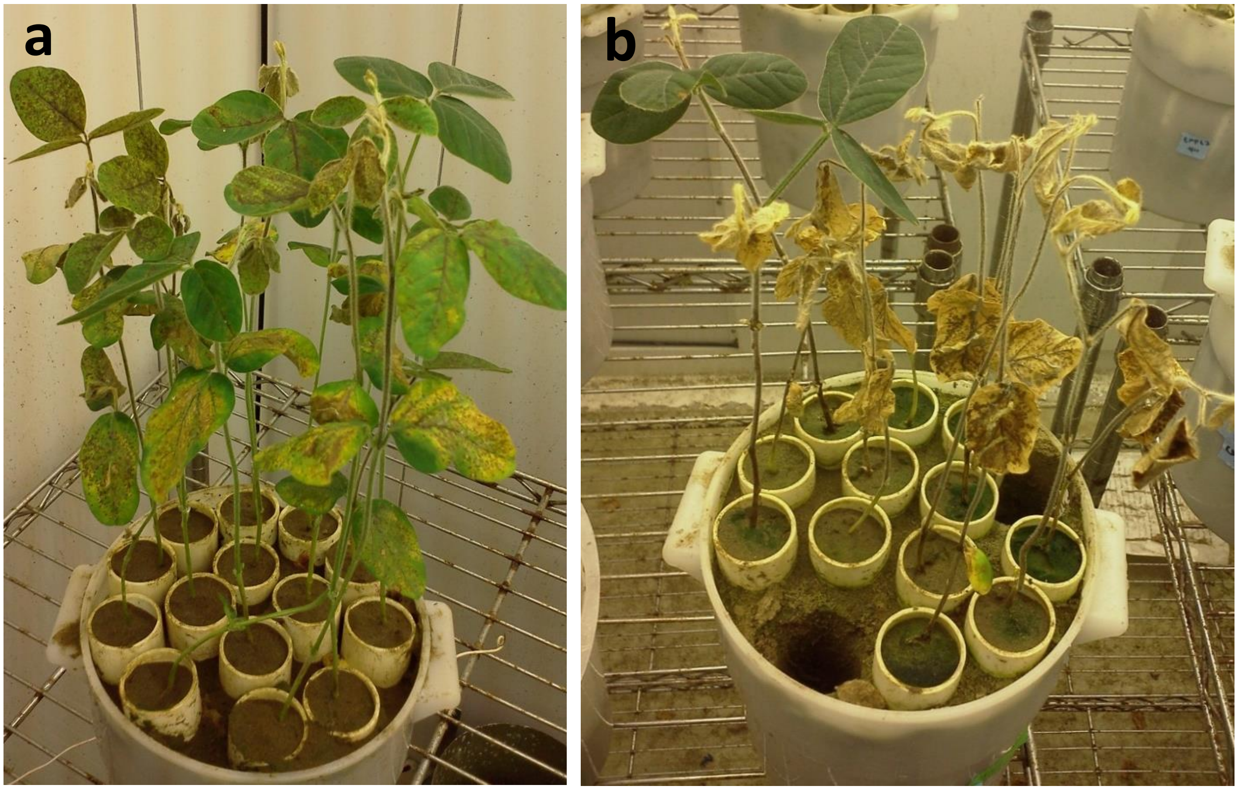

Supplementary Figure 2. Plant growth of RIL ExF67 (*GmSNAP18*$^{+}$/*GmSHMT08*$^{+}$) inoculated with BPMV-Forrest *GmSNAP18*. (**a**) 16 days post VIGS infection. (**b**) 30 days post VIGS infection.

a

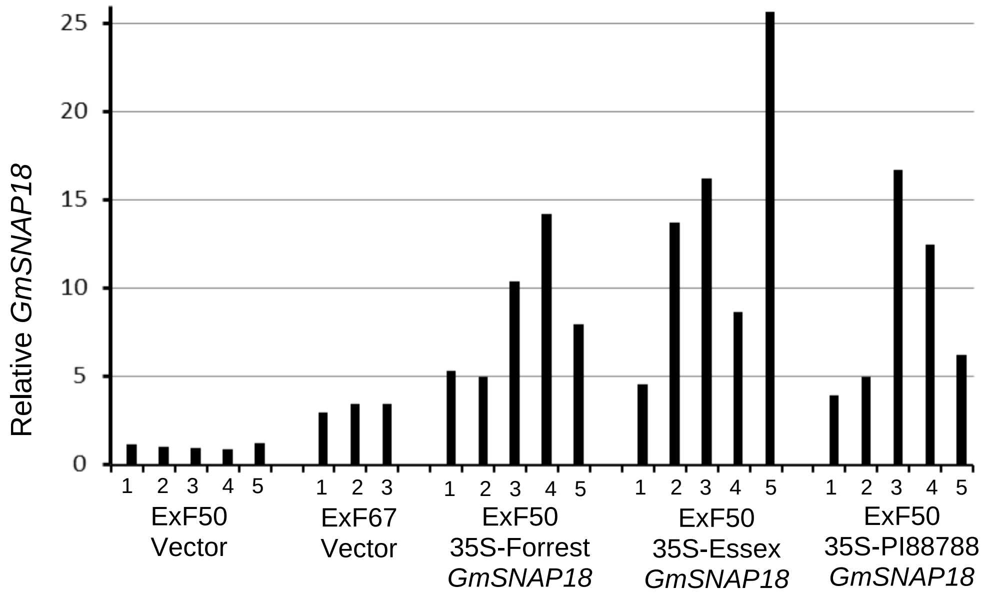

b

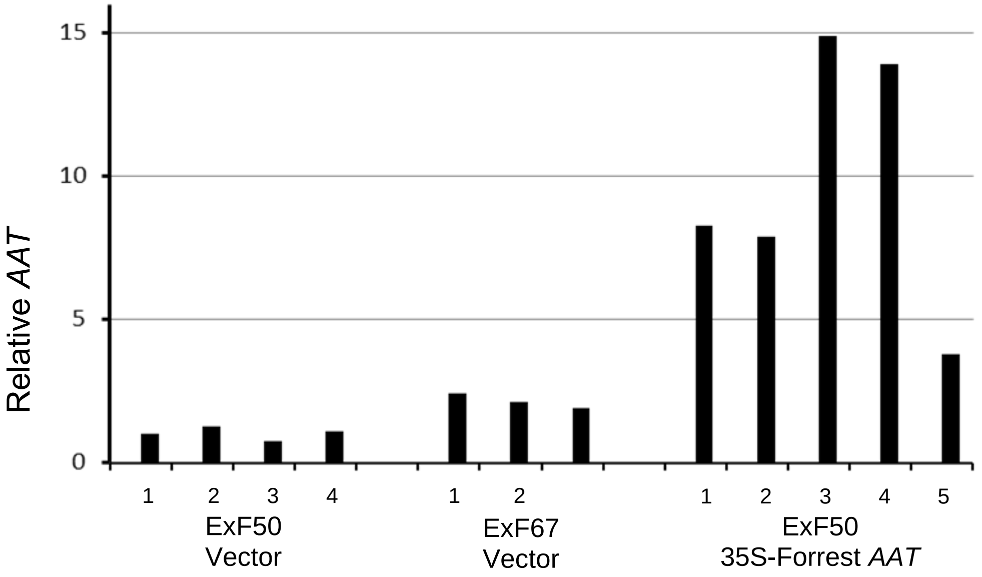

Supplementary Figure 3. Expression analysis of transgenic hairy roots. (**a**) Expression of Forrest *GmSNAP18* in ExF50 (*GmSNAP18*$^{-}$/*GmSHMT08*$^{+}$) *GmSNAP18* transgenic hairy roots. Numbers denote independent lines used for the analysis. Data is relative to the Forrest *GmSNAP18* expression in one of the ExF50 vector control roots. (**b**) Expression analysis of *AAT* in ExF50 *AAT* transgenic hairy roots. Numbers denote independent lines used for the analysis. Data is relative to the expression in one of the ExF50 vector control roots. ExF67 (*GmSNAP18*$^{+}$/*GmSHMT08*$^{+}$) was taken as the control. The qPCR primers used for the expression analysis are listed in the primer table (**Supplementary Table 1**).

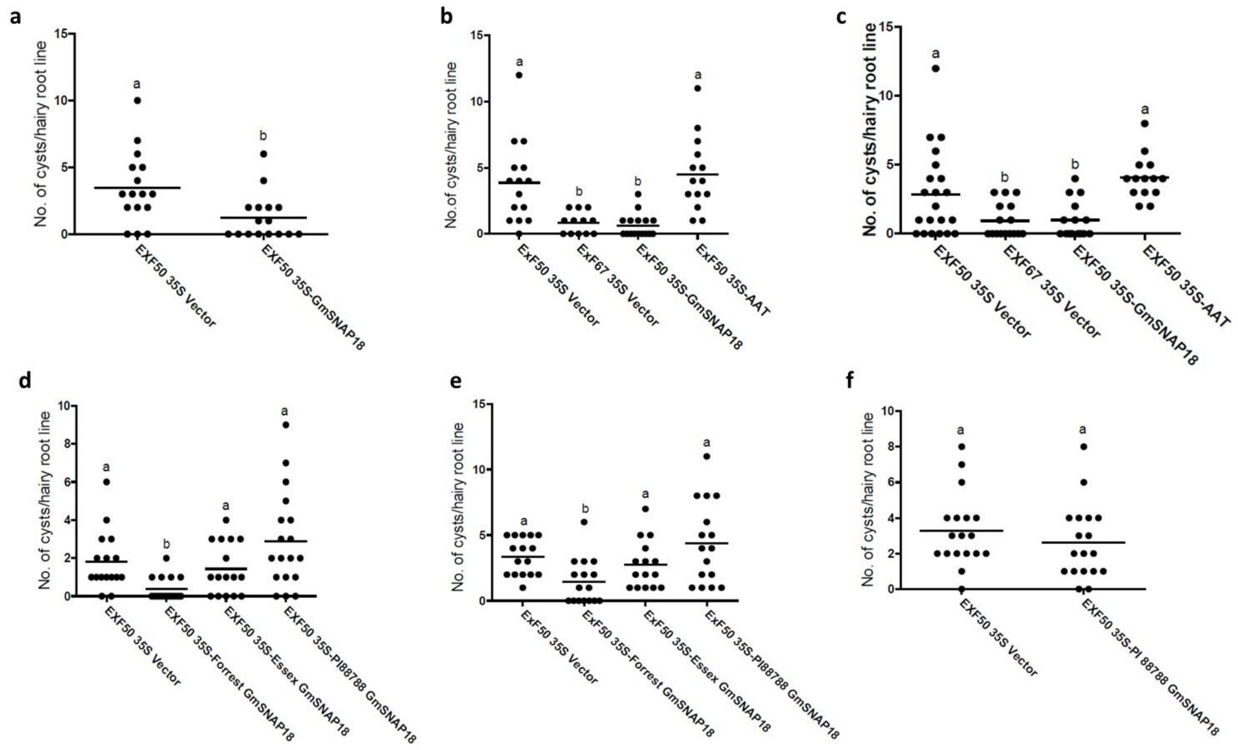

Supplementary Figure 4. Replicate experimental data for nematode infection assays. (**a**) SCN infection of RIL ExF50 transgenic hairy roots expressing Forrest *GmSNAP18*. (**b**) and (**c**) SCN infection of RIL ExF50 transgenic hairy roots expressing Forrest *GmSNAP18* or *AAT*. (**d**), (**e**) and (**f**) SCN infection of RIL ExF50 transgenic hairy roots expressing *GmSNAP18* of Forrest, Essex or PI 88788. *GmSNAP18* refers to Forrest-*GmSNAP18*, if not indicated otherwise. Significantly different experimental results obtained by an unpaired *t*-test are denoted by different letters ($P<0.05$, range from 0.04 to 0.0004). Each dot denotes cyst number from an independent hairy line ($n>=12$).

Supplementary Table 1 The primers used in this study except the primers employed for RSE-Seq. Some of primers referred to the Supplementary References, and the left were designed and developed in this study.

I. Primers for screening of *r hg1-a* recombinants and genotyping of *Rhg4*

rhg1	
Sat_210[4]	Forward: GCGCCAGCAACAAAGTTCCTGACAAA
	Reverse: GCGCATGCAAATGAAATAATAA
Satt309[4]	Forward: GCGCCTTCAAATTGGCGTCTT
	Reverse: GCGCCTTAAATAAAACCCGAAACT
SIUC-SAT143[1]	Forward: TGTTACTTAGTAATTATGAAG
	Reverse: AATAATGATTTGTTGATCGAT
Rhg4	
Satt632[4]	Forward: GGGCTATGAAGGGAATGGAAAGGA
	Reverse: CCCATATTGAAGATTTGAAGTAAT
Sat_162[4]	Forward: GCGTGGTTTTTCGCTGGATATA
	Reverse: GCGCATTTCGTAACATATTTTTCAC
GMES6186[3]	Forward: AGCGGGAATTGAAGGTTTTT
	Reverse: GGAATCTCATCTGAAAATAATGGA

II. Primers for high density genetic mapping of *r hg1-a* recombinants and haplotyping of soybean lines

rhg1	
Sat_210[4]	Forward: GCGCCAGCAACAAAGTTCCTGACAAA
	Reverse: GCGCATGCAAATGAAATAATAA
5601	Forward: TTACTTTTGGTCAGCATTTTGGC
	Reverse: TATTGTTGATATATTATATTGTCC
5701	Forward: ACCCTTTTTGCAGTATTTATGC
	Reverse: CTAGGTAACTCTTTTAGCCGTGA
580	Forward: GACCAAGGTGAAAAGCAAAGCA
	Reverse: ATAGTAGGCTGCCAGGCACCG
590	Forward: ATGGCCGATCAGTTATCGAAGGG
	Reverse: ACCCAGGAGAAGAGATATCAATA
600	Forward: GAAATGCATGGTTTGAAGTAAAC
	Reverse: GACAGAGTTAATGTCTATAGTAT
610	Forward: GTTTGGTTTGGTTACTTGAAAATC
	Reverse: GGCATCATCACAACCCAACT
10	Forward: CTCTAACCATTTCGACTTGTTGC
	Reverse: ATGAGTCTATGACCATACCCTT
12	Forward: CAAAAGTCTGACTTAAAAGTCTC
	Reverse: GTCGTCTGTAGACTGATACAG
122452	Forward: CGCAAACTTTATTGTGTTCCTAGTC

	Reverse: TGACCCACCTGATAAAATGTAGT
TMD1	Forward: AAACTAGTCCATTTTGATGGA
	Reverse: AGCATTTGTATTCTCATCAAG
Satt309[4]	Forward: GCGCCTTCAAATTGGCGTCTT
	Reverse: GCGCCTTAAATAAAACCCGAAACT
375912	Forward: TGCTCTCAACTCATGCTCCTAC
	Reverse: ACTTGGTGCTTCTTGGATTGTG
122592	Forward: GCCGTCTTCTTTTTCTCTTTTCACTTTCTAA
	Reverse: TGTTGGCTTTTCCTTTTCATTTATCT
120	Forward: TTTGGTTGATGTTGTTGCATC
	Reverse: GGGAAATAAGAAAAACAATGCT
SIUC-SAT143[1]	Forward: TGTTACTTAGTAATTATGAAG
	Reverse: AATAATGATTTGTTGATCGAT
SIUC-SAT185	Forward: CAGAGTCAAGTTTGAACTTCAT
	Reverse: AATCAAGTGATCATTGTACTG
375752	Forward: GCTGCCTCAATTCAACAATCC
	Reverse: TTGGTGTCATGTCTTGCTCTC
***GmSHMT08* sequencing**	
SHMT[1]	Forward: ACAACACTCTCTCTTCTCGC
	Reverse: CAGATTATGAGTTTTGGCCTG
SHMT2[1]	Forward: CAGGCCAAAACTCATAATCTG
	Reverse: CGTTACCAATTCGCACTCCA
SHMT3 [1]	Forward: TAATTTTGGTTGGAGAACAATG
	Reverse: CTAATCCTTGTACTTCATTTC
***GmSNAP18* sequencing**	
SNAP1	Forward: CGCTTATGAATCTTCTTCTTCTTC
	Reverse: GCATGTAGTGGTAACAACTGAGAC
SNAP2	Forward: GGTTTTGGAGTGGGCTGAATC
	Reverse: ACCCAGGAGAAGAGATATCAATA
SNAP3	Forward: GCAGGAGCAAATTATTTTGCTGTC
	Reverse: GAATTTGATGACACGTACAATAAATG
SNAP4	Forward: CACTGTGTAAAGTTAATTTTTTTGCTTAC
	Reverse: CCAATTCAATTAAAACCAAAGCAGG

III. Primers for cDNA sequencing

Glyma18g02570-cDNA	Forward: ATGCGCGCCCTAGCAGCTCA
	Reverse: TCATGATGGCACATCATATCTGAG
Glyma18g02580-cDNA	Forward: ATGTCTCCGGCCGCCGGAGA
	Reverse: TCATGACTTGCTACTAAAAGCATTAT
Glyma18g02590-cDNA	Forward: CGCTTATGAATCTTCTTCTTCTTC
	Reverse: CCAATTCAATTAAAACCAAAGCAGG

IV. Primers for subcloning

GmSNAP18-F (Forrest/Essex/PI88788)	AAAAAAGCAGGCTAT ATG GCC GAT CAG TTA TCG AA
GmSNAP18-Forrest-R	AAGAAAGCTGGGTCTCA AGT AAT AAC CTC ATA CTC CTC
GmSNAP18-PI88788-R	AAGAAAGCTGGGTC TCA AGT AAT AGC CTC ATG CTG CTC
GmSNAP18-Essex-R	AAGAAAGCTGGGTCTCA AGT AAG ATC ATC CTC CTC
GmAAT-F	AAAAAAGCAGGCTTA GAAGGAGATAGAACCATGTCTCCGGCCGCCGGAGT
GmAAT-R	AAGAAAGCTGGGTC TTA TGA CTT GCT ACT AAA AGC ATT
attB1-F	GGGGACAAGTTTGTACAAAAAAGCAGGCT
attB2-R	GGGGACCACTTTGTACAAGAAAGCTGGGT

V. Primers for VIGS

GmSNAPVIGS-F	CGCGGATCCCTCGAGATCCAACATTTTCAGGAACACG
GmSNAPVIGS-R	CGCGGATCCCCAGTTCCTGATATCGTTCTAA

VI. Primers for qRT-PCR

GmSNAP18-RT-F	ACAAGGCTGGAGCGACATAC
GmSNAP18-RT-R	AGCAATGTGCAGCATCGACA
qGm18AAT-F	GGTTTGGAATTCACTGGTGGAA
qGm18AAT-R	CAATACCAATGGAAGCATAACAAAGA
GmUbiquitin-F	GTGTAATGTTGGATGTGTTCCC
GmUbiquitin-R	ACACAATTGAGTTCAACACAAACCG

Supplementary Table 2 Direct identification of *rhg1* candidate gene *GmSNAP18* conferring resistance to SCN by analysis of the polymorphisms possibly associated with the alleles underlying resistance to SCN. The results indicate that gene Glyma18g02590 (*GmSNAP18*) is the candidate gene most possibly associated with the resistance to SCN at *rhg1*, in which those 6 SNPs (C163208A, C163225G, G164965C, G164968T, G164968C and C164974A) and 4 insertions (A164972AGGT and A164972AGGC) are all located within the exons, leading to the amino acid (AA) changes of predicted GmSNAP18 protein.

Gene	SNPs/Insertions	Total AA changes	AA (nucleotide) changes in Forrest/Peking	AA (nucleotide) changes in PI88788
Glyma18g02353	1	1	1	-
Glyma18g02420	1	1	1	-
Glyma18g02450	3	3	3	-
Glyma18g02520	1	1	-	1
**Glyma18g02590 (*GmSNAP18*)**	**10**	**9**	**3+2<br>D208E (C163225G)<br><br>D286Y (G164968T)<br>D287E and -288V (A164972AGGT)<br>L289I (C164974A)**	**4+2<br>Q203K (C163208A)<br>E285Q (G164965C)<br>D286H (G164968C)<br>D287E and -288A (A164972AGGC)<br>L289I (C164974A)**
Glyma18g02650	1	1	1	-
Glyma18g02660	1	1	1	-
Glyma18g02681	1	1	1	-
Glyma18g02690	3	3	3	-
Glyma18g02700	1	1	1	-
Glyma18g02720	2	2	-	2
Glyma18g02741	1	1	-	1

Supplementary Table 3 The primers specific for the enrichment of the 300 kb regional genomic DNA segment at *rhg1* by RSE.

No.	Primers (5'>3')	No.	Primers (5'>3')
Gm18-1	ctctatcgactctcagtctc	Gm18-31	cctggaaacaagccatgttc
Gm18-2	acatacaatataagcctgcgaag	Gm18-32	cgtgacacacctctgtctaa
Gm18-3	cgccttgtgcaaaagaagat	Gm18-33	cccaaactgaaagcgtgaag
Gm18-4	actaacgccacttaccacca	Gm18-34	gtgtaatgaatggacggtcc
Gm18-5	gtgactacgatgtcactgac	Gm18-35	agagtattatgaacggcgtct
Gm18-6	ccaattcccacctcaggtaa	Gm18-36	gacttcgcaaatccaagacg
Gm18-7	ccagactcaagtctaagctc	Gm18-37	ggggtgtcaagacaatctag
Gm18-8	gaacgacactgatatgctgg	Gm18-38	gacacaaaaggcttgtttcc
Gm18-9	ctagtgtctgcatcatcaca	Gm18-39	gcagttctttagctgttctg
Gm18-10	ccgaagttaggaaagaaggg	Gm18-40	cctgaatttcttggtaaagcaag
Gm18-11	gaactcttgaatgcttcctgca	Gm18-41	gcagctattccagttctgtt
Gm18-12	gctctataaatagagcattcgtagtct	Gm18-42	ggggagtaaaacacctcctc
Gm18-13	gttggtccctagaatatctga	Gm18-43	ccttatcatcaaccgcaaca
Gm18-14	gcatcggaatgaacgctaac	Gm18-44	gaacagcacggctatcatca
Gm18-15	cgaggatcaggatgattttg	Gm18-45	ctcactttggtagtagtctc
Gm18-16	cctgtgtagtaactagtaac	Gm18-46	gttagccatggatcgaatgaag
Gm18-17	cacgtacacccatattctga	Gm18-47	gtgtctcagaacccagttag
Gm18-18	agtaggggtacgggtatgag	Gm18-48	aacggataacaagacagaaagaa
Gm18-19	ctttcgttttcttagcacatgtctt	Gm18-49	gttacacgagtcaggaccat
Gm18-20	gcttctagaagtaaaactaacga	Gm18-50	aacaaggttgcctgcaagtc
Gm18-21	cattcaggcatactaaacttgttgc	Gm18-51	ctttgcattagcgagaaaatttgtg
Gm18-22	ctcaaggaggaggaagtgat	Gm18-52	tcatcttcctgatggaggct
Gm18-23	tttatcatggcctaggcctacca	Gm18-53	ccggacttaggataactaaaacaag
Gm18-24	ccatcacggcatctttaaatttatc	Gm18-54	catgggaggaatgaaacgctgga
Gm18-25	ccgtatgagacccgcatatt	Gm18-55	cgtaagccttactttataccatac
Gm18-26	tgatgatttttagcggtcag	Gm18-56	gggcctagctaggcattata
Gm18-27	tgcgttaagagatgatccag	Gm18-57	ccgaggtggactttgattcttct
Gm18-28	acatcaggcaattcccgtag	Gm18-58	gtccctcaaaggcattcttt
Gm18-29	cagcattcctcatatggagtg	Gm18-59	cgagtggactaagttcctta
Gm18-30	cattaaataggtcgcctaca	Gm18-60	tccgtgaatgattgacaagg

Supplementary References

1. Liu, S., Kandoth, P.K., Warren, S.D., Yechel, G., Heinz, R., Alden, J., Yang, C., Jamai, A., Ei-Mellouki, T., Juvale, P.S., Hill, J., Baum, T.J., Cianzio, S., Whitham, S.A., Korkin, D., Mitchum, M.G. & Meksem, K. A soybean cyst nematode resistance gene points to a new mechanism of plant resistance to pathogens. *Nature* **492**, 256-260 (2012).

2. Hyten, D.L., Choi, I.Y., Song, Q., Shoemaker, R.C., Nelson R.L., Costa, J.M., Specht, J.E. & Cregan, P.B. Highly variable patterns of linkage disequilibrium in multiple soybean populations. *Genetics* **175**, 1937-1944 (2007).

3. Hwang T.Y., Sayama, T., Takahashi, M., Takada, Y., Nakamoto, Y., Funatsuki, H., Hisano, H., Sasamoto, S., Sato, S., Tabata, S., Kono, I., Hoshi, M., Hanawa, M., Yano, C., Xia, Z., Harada, K., Kitamura, K. & Ishimoto, M. High-density integrated linkage map based on SSR markers in soybean. *DNA Res.* **16**, 213-225 (2009).

4. http://soybase.org
